# A corpus-based study of metadiscourse features in Chinese-English simultaneous interpreting

**DOI:** 10.3389/fpsyg.2023.1269669

**Published:** 2023-11-09

**Authors:** Wen Ren, Lu Wang

**Affiliations:** Graduate School of Translation and Interpretation, Beijing Foreign Studies University, Beijing, China

**Keywords:** Chinese-English simultaneous interpreting, metadiscourse features/markers, interpreting corpus, metacognition, professional interpreters

## Abstract

Metadiscourse markers have been observed to be frequently employed by simultaneous interpreters as interpreting strategies in the process of interpretation. However, the realm of inquiry into this phenomenon remains relatively underexplored. This study aims to rectify this gap by conducting a systematic analysis of metadiscourse features in the context of Chinese-English simultaneous interpreting. The analytical framework for this study is rooted in Hyland’s interpersonal model of metadiscourse. By comparing the frequency and distribution of various metadiscourse markers in the source language (Chinese), target language (English), and original English speeches in simultaneous interpreting events, the research seeks to offer a quantitative description, qualitative analysis, and explanation of the causes of these metadiscourse features. To facilitate this analysis, the authors have compiled a Chinese-English Simultaneous Interpreting Parallel Corpus and a Comparable Original English Corpus based on ten international economic conferences, totaling 161,068 tokens. The findings reveal significant differences in interlingual and intralingual comparisons. Professional interpreters demonstrate a strong awareness of metadiscourse by employing various “metadiscourse interpreting strategies,” including explicitation/simplification, emphasis/weakening, and visibility/invisibility. Moreover, interpreted English displays distinct metadiscourse features including explicitation of textual logic, objective modal tendency, and audience orientation, as compared to the original English speeches. The observed differences in the study can be attributed to a range of factors, including contextual factors, source language features, and the cognitive psychology of the interpreter. This study provides an in-depth analysis of metadiscourse characteristics in Chinese-English simultaneous interpreting, contributing to fine-grained inquiries into our understanding of the linguistic features of interpreted texts within the context of corpus-based interpreting studies.

## 1. Introduction

Interpreted texts, as a form of translated text, exhibit distinctive linguistic characteristics known as “interpretese” (Shlesinger, 2008). An examination of these features can enrich our understanding of interpreting from both product and process perspectives (Bendazzoli, 2018). Among the numerous linguistic features in discourse, metadiscourse serves not only as a primary means for organizing discourse, explicitating discourse intentions, and enhancing audience engagement (Hyland, 2019, pp. 217–218) but also as a linguistic reflection of metacognition (Shi, 2022, p. 59). In interpreting, metadiscourse assumes a pivotal role in ensuring discourse coherence, fostering interpersonal interaction, and facilitating effective communication (Diriker, 2009; Lee, 2015; Fu, 2017). Recent years have witnessed numerous researchers exploring metadiscourse in translation across different text types, language pairs, and research topics (Peterlin, 2008; Kim, 2011; Gholami et al., 2014; Farahani and Dastjerdi, 2019; Afzaal et al., 2022). However, investigations into metadiscourse in interpreting remain relatively limited (Diriker, 2009; Lee, 2015; Fu, 2017). Academic inquiries in Chinese-English interpreting have predominantly focused on political discourse in consecutive interpreting (Fu, 2017; Gu and Tipton, 2020; Gu and Wang, 2021), with much less attention directed towards simultaneous interpreting (Sun, 2018). Besides, most existing research endeavors have primarily concentrated on a restricted selection of metadiscourse markers, leading to an incomplete grasp of metadiscourse within the sphere of simultaneous interpreting.

In written communication, metadiscourse markers serve as useful tools for writers, aiding in the coherent organization of texts and facilitating the conveyance of attributes such as personality, credibility, reader sensitivity and relationship to the message (Hyland, 2019, p. 71). Within the domain of interpreting, metadiscourse is seen as a constellation of essential grammatical resources, upon which interpreters rely to structure discourse, guide the audience through its intricacies, evaluate the propositions embedded therein on behalf of the speaker, and engage with the audience to optimize the transmission of the speaker’s communicative intent (Fu, 2017). Metadiscourse markers have also been observed to be frequently used by simultaneous interpreters to manage and regulate interaction between speakers and listeners (Diriker, 2004, p. 168). Given the significance of metadiscourse in interpreting discourse evidenced above, this study undertakes an empirical trajectory by harnessing a corpus-based method to examine metadiscourse features in Chinese-English simultaneous interpreting. To this end, we have built a Chinese-English Simultaneous Interpreting Parallel Corpus and a Comparable Original English Corpus based on ten economic conferences, totaling 161,068 tokens. In its conceptual underpinning, this study aligns itself with Hyland’s interpersonal model of metadiscourse (Hyland, 2019, p. 58). With this theoretical framework, the study further employs interlingual and intralingual comparisons facilitated by the integration of parallel and comparable corpora (Laviosa, 2012) to provide a comprehensive analysis of metadiscourse features in Chinese-English simultaneous interpreting.

## 2. Literature review

In this part, we first look at the various definitions and classifications of metadiscourse provided by scholars, in particular by Hyland. Then we examine the existing discussion of the relationship between metadiscourse and metacognition before briefly reviewing the previous research on metadiscourse in translation and interpreting.

### 2.1. Definitions of metadiscourse

In 1959, Harris introduced the notion of metadiscourse, which refers to linguistic devices used by writers or speakers to guide a receiver’s perception of a text (Hyland, 2019, p. 1). This concept provides a novel perspective for analyzing language use. Williams (1981) and Crismore (1983) distinguished metadiscourse from propositional content by delineating its various functions, thereby facilitating the application of metadiscourse in discourse research. According to Hyland and Tse (2004), metadiscourse can be narrowly understood as features of textual organization, while more broadly, it can be seen as all the linguistic and rhetorical manifestations employed by authors.

This more extensive interpretation aligns with Hyland’s subsequent definition, wherein he posited metadiscourse as “the cover term for self-reflective expressions used to negotiate interactional meanings in a text, helping the writer (or speaker) to express a viewpoint and engage with readers as members of a particular community” (Hyland, 2005, p. 37). This definition explicitly indicates that any form of expression does not merely convey information but also encompasses the emotions, attitudes, and values of the writer (or speaker) and their communicative intentions when interacting with the reader (or audience). Specifically, writers or speakers can significantly influence the readers’ or audiences’ interpretation of the text by adopting various categories of metadiscourse.

### 2.2. Classifications of metadiscourse

Over the past three decades, scholars have proffered various metadiscourse classification approaches. Vande Kopple (1985) initially partitioned metadiscourse into seven types from a functional perspective. Later, influenced by Halliday’s Systemic Functional Grammar theory and its three metafunctions of language, Vande Kopple and Shoemaker (1988) undertook a reclassification of the previous seven typologies into textual and interpersonal metadiscourse. Crismore et al. (1993), in a revisitation of Vande Kopple’s (1985) classification, enacted refinements, rendering it more detailed and hierarchical in structure. They modified the subcategories and integrated punctuations to make the metadiscourse framework more comprehensive, affording a heightened range of inclusivity, and augmenting its explanatory potency in written language. Among the diverse metadiscourse classification approaches, Hyland’s (2005, 2019) interpersonal metadiscourse model has exerted the most profound influence. This model encapsulates the metadiscourse functions delineated by Vande Kopple and Crismore et al., and demonstrates explicitness, comprehensiveness, and lucidity (Abdi et al., 2010). It addresses the problems in prior classifications, characterized by overlapping categorizations and nebulous boundaries. Each category within this model is provided with almost unambiguous definitions and exemplifications, thereby demonstrating a high degree of operability.

### 2.3. Relationship between metadiscourse and metacognition

Metadiscourse and metacognition are two pivotal concepts in linguistics and cognitive science, respectively. Previous studies have examined the relationship between these two concepts from various perspectives. Mauranen (2023, p. 5) highlighted that metadiscourse is part of discourse, while metacognition is part of cognition, and they both stand in a “meta” relationship within their respective fields. Tang (2021, p. 11) also pointed out that metadiscourse and metacognition are both used as self-referential tools to reflexively engage with one’s own thoughts and speech.

Although they share some common features, these two concepts diverge in their focal points. Metadiscourse is a broad term inclusive of the self-reflective expressions used to negotiate interactional meanings in a text (Hyland, 2019, p. 43), while metacognition refers to the ability to consciously regulate, monitor, and harmonize cognitive functions, representing knowledge and cognition about cognitive phenomena (Flavell, 1976, 1979). Crismore (1989) held that discourse consists of primary discourse and metadiscourse. The generation and comprehension of primary discourse constitute the subject or object of the cognitive process, while metacognition regulates and monitors the object through metadiscourse, collectively contributing to effective communication (Li, 2003, p. 30). Shi (2022, p. 59) further proposed that speakers employ metacognition to organize, monitor, and adjust their discourse according to the listener’s understanding, with metadiscourse serving as the linguistic representation of metacognition.

Moreover, several studies have highlighted the vital role of metacognition and metadiscourse in interpreting, particularly in the interpreting process and interpreting competence. Arumí and Esteve (2006) investigated the function of metacognition in the interpreting process and found that metacognitive guides can foster interpreters’ self-reflection, self-regulation, and autonomous learning. Moser-Mercer (2008) suggested that learners with meta-cognitive abilities can enhance their acquisition of interpreting skill by using techniques such as “self-talk” or “journaling” to understand and encode the process, practice skills, and obtain feedback from the learning environment. In terms of the functions of metadiscourse in interpreting, in addition to regulating interaction (Diriker, 2004) as discussed in the introduction, metadiscourse also serves to ensure accuracy (Lee, 2015), reconstruct intertextual and intratextual coherence (Fu, 2017), and enhance logical cohesion (Sun, 2018). Therefore, it can be assumed that metadiscourse and metacognition complement each other and work collaboratively throughout the interpreting process. Metadiscourse indicates interpreters’ capacities to modulate cognitive processes during interpreting, while metacognition governs and optimizes interpreters’ cognitive operations at a higher level. An investigation into metadiscourse in interpreting can provide valuable insights into the cognitive processes involved, with significant implications for both theoretical understanding and practical applications in the field.

### 2.4. Metadiscourse in translation and interpreting

Metadiscourse markers are ubiquitous across languages (Hyland et al., 2022) and have progressively woven themselves into the tapestry of translation and interpreting research. In the metadiscourse inquiry pertaining to translation, scholars have conducted comprehensive investigations that span the spectrum of text types, languages, translating subjects, and research topics. Firstly, scholars have extensively explored metadiscourse in translation across text types, including academic discourse (Peterlin, 2008; Liu et al., 2022), business texts (Kim, 2011), medical literature (Gholami et al., 2014), news editorials (Shin, 2015; Kim, 2020), and hotel websites (Suau Jimenez, 2015). Moreover, these investigative foray spans various languages, including English, Slovenian, French, Korean, Persian, Arabic, Spanish, and Chinese. Furthermore, inquiries have delved into the use of metadiscourse markers wielded by different translating subjects. For instance, Afzaal et al. (2022) compared metadiscourse used by machine and human translators. Williams (2010) assessed metadiscourse translation performance by student translators. The scope of research topics is also wide-ranging, covering aspects such as the interplay between information senders and receivers in audiovisual translation (Farahani and Kazemian, 2021), the impact of metadiscourse on text politeness (Kim, 2011), the function of metadiscourse in literary translation (Fathi, 2019), comparative analyses of metadiscourse markers in different translated versions of the same classical text (Farahani and Dastjerdi, 2019; Savaskan, 2021), and the cross-cultural factors behind metadiscourse strategies used in the translation process (Giordano and Marongiu, 2022).

Within the domain of metadiscourse research in interpreting studies, prominent themes include discourse coherence, interpreting strategies, the interpreter’s professional image, and the various roles of interpreters. Fu (2017) investigated the relationship between metadiscourse and coherence in interpreting, emphasizing the crucial role of metadiscourse in reconstructing both intertextual and intratextual coherence. Lee (2015) examined the use of interactional metadiscourse in court interpreting, demonstrating its significant role in managing stance, facework, rapport, and ensuring the accuracy of interpreting. Moreover, additional scholarly efforts have ventured into the inquiry of the influence of specific metadiscourse markers on the professional identity of simultaneous conference interpreters (Diriker, 2004, 2009) and interpreters’ mediation in political discourse (Gu and Tipton, 2020).

Compared to extensive research dedicated to metadiscourse in translation, metadiscourse studies in interpreting remain relatively limited. Most existing studies have focused on selected metadiscourse markers, thereby not fully grasping the full scope of this phenomenon. Furthermore, the corpora employed for investigation have mostly been circumscribed to political or legal topics. Methodologically, scholars have predominantly adopted interlingual comparisons, while the application of intralingual or multidimensional comparative approaches to investigate metadiscourse features in interpreting is notably restricted. Regarding language pairs, research has primarily centered on specific combinations, such as Chinese-English, Korean-English, and English-Turkish. In Chinese-English interpreting research, emphasis has been placed on the examination of consecutive interpreting of political discourse, giving much less attention to simultaneous interpreting discourse. Consequently, the systematic analysis of metadiscourse features in Chinese-English simultaneous interpreting remains underexplored. This notable gap underscores the significance of venturing into this hitherto less charted territory, as it unfolds as an important yet somewhat overlooked avenue for scholarly inquiry.

Since the 1990s, corpus-based research methods have been employed in translation (Baker, 1993) and interpreting studies (Shlesinger, 1998), significantly broadening the scope and depth of the research in these fields. In recent years, corpus-based methods have also gained widespread attention and application in metadiscourse research (Hyland and Jiang, 2022). The employment of corpus-based methods to examine metadiscourse in interpreting holds particular significance, as has been evidenced by Fu’s (2017) research. However, corpus-based studies on metadiscourse in simultaneous interpreting have been scarce, accentuating the importance of the current research.

## 3. Theoretical framework

This research adopts Hyland’s (2019, p. 58) interpersonal model of metadiscourse as its theoretical framework to systematically analyze metadiscourse features in simultaneous interpreting. This is because Hyland’s model is believed to be a simple, clear and inclusive model that builds upon previous taxonomies (Abdi et al., 2010, p. 2), which endows it with applicability across various contexts. It has garnered substantial attention and utilization within academic discourse since the mid-2000s (Wei et al., 2016, p. 201).

As metadiscourse analysis involves taking a functional approach to texts, scholars in this area have often turned to the Systemic Functional Theory of language for insights and theoretical underpinning (Hyland, 2019, p. 30). Halliday (1973) pioneered classifying language into three metafunctions: ideational, interpersonal, and textual. The ideational function corresponds to propositional discourse, while the interpersonal and textual functions fall under metadiscourse. While most scholars based their categorization of metadiscourse “as either performing a textual function by organizing a coherent discourse, or performing an interpersonal function by conveying the writer’s attitudes to the text,” Hyland (2019, p. 30) offered a different perspective on the textual function. Hyland (2019, pp. 50–51) argued that textual devices not only structure text propositions but also engage readers interpersonally, subsuming the textual within the interpersonal. As Hyland (2019, p. 51) stated, the “so-called ‘textual’ devices organize texts as propositions by relating statements about the world and as metadiscourse by relating statements to readers; they do not function independently of these two functions.” Thus, Hyland (2019, p. 53) posited textual metadiscourse as another aspect of the interpersonal features of a text, construing both propositional and interpersonal aspects of texts into a coherent whole.

Building on earlier classifications of metadiscourse (Vande Kopple, 1985; Crismore et al., 1993) and employing Thompson and Thetela’s (1995) distinction between interactive and interactional resources, Hyland’s model (Hyland and Tse, 2004; Hyland, 2005, 2019) is also composed of these two dimensions. The interactive dimension facilitates cohesive discourse organization, catering to the needs of readers/listeners, including transitions, frame markers, endophoric markers, evidentials, and code glosses. On the other hand, the interactional dimension conveys the author/speaker’s presence and the degree of collaboration with readers/listeners in co-constructing the text, involving hedges, boosters, attitude markers, self-mentions, and engagement markers. Although Hyland introduced certain adjustments or changes in expression across different versions of the interpersonal model of metadiscourse over time (Hyland and Tse, 2004; Hyland, 2005, p. 49; Hyland, 2019, p. 58), the core categories, functions, and illustrative examples of metadiscourse have remained largely consistent. In the current study, we align with his most recent version (see Table 1; Hyland, 2019, p. 58) for our analysis.

**Table 1:** An interpersonal model of metadiscourse (Hyland, 2019, p. 58).

Category	Function	Examples
*Interactive*	*Help to guide the reader through the text*	*Resources*
Transitions	express relations between main clauses	in addition; but; thus; and
Frame markers	refer to discourse acts, sequences or stages	finally; to conclude; my purpose is
Endophoric markers	refer to information in other parts of the text	noted above; see Fig; in section 2
Evidentials	refer to information from other texts	according to X; Z states
Code glosses	elaborate propositional meanings	namely; e.g.; such as; in other words
*Interactional*	*Involve the reader in the text*	*Resources*
Hedges	withhold commitment and open dialogue	might; perhaps; possible; about
Boosters	emphasize certainty or close dialogue	in fact; definitely; it is clear that
Attitude markers	express writer’s attitude to proposition	unfortunately; I agree; surprisingly
Self-mentions	explicit reference to author(s)	I; we; my; me; our
Engagement markers	explicitly build relationship with reader	consider; note; you can see that

Although originally developed for the scrutiny of academic writing, Hyland’s model has extended beyond its initial domain in recent years. Numerous studies have applied Hyland’s model to the research of spoken metadiscourse (Kuhi et al., 2014; Kahkesh and Alipour, 2017; Farahani, 2020; Farahani and Kazemian, 2021). Furthermore, Fu (2017) expanded its application to the analysis of interpreted texts in Chinese-English consecutive interpreting. Overall, it has become the most frequently cited and extensively utilized analytical model for metadiscourse analysis since the mid-2000s (Wei et al., 2016, p. 201). These preceding studies have affirmed the suitability, relevance and analytical potency of Hyland’s model in its application in the investigation of metadiscourse in simultaneous interpreting, laying a strong foundation for the current research. Hence, it is adopted as the theoretical framework for the analysis in this study.

## 4. Research questions and research methods

Grounded in the theoretical underpinnings of Hyland’s interpersonal model of metadiscourse, this study seeks to provide a comprehensive analysis of metadiscourse features in Chinese-English simultaneous interpreting. This investigation employs quantitative description, qualitative interpretation, and causal explanation, utilizing resources from a Chinese-English Simultaneous Interpreting Parallel Corpus and a Comparable Original English Corpus.

### 4.1. Research questions

To achieve the research objective, this study addresses the following questions:

1. Are there significant differences in the distribution of metadiscourse markers between the source language (Chinese) and target language (English) in simultaneous interpreting?
2. Are there significant differences in the distribution of metadiscourse markers between the target language (English) in simultaneous interpreting and original English?
3. What factors contribute to the differences in metadiscourse features in these two comparison modes?

### 4.2. Research methods

This study employs a mixed-methods approach, integrating quantitative and qualitative analysis. In the quantitative data analysis stage, the primary focus is on examining the frequency and distribution patterns of metadiscourse markers across the corpora to ascertain whether significant differences exist among the source language (Chinese), target language (English), and original English. In the qualitative research stage, we examine the functions of metadiscourse markers in various contexts, utilizing representative examples to provide a comprehensive interpretation.

**Figure 1:**
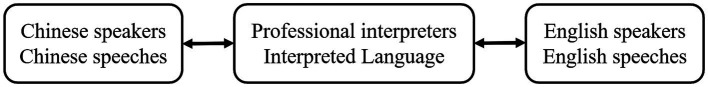
Composite comparative mode of metadiscourse in SI.

To facilitate this analysis, we have compiled a Chinese-English Simultaneous Interpreting Parallel Corpus (comprising a Source Language Corpus and a Target Language Corpus) and a Comparable Original English Corpus. We employ a composite comparative model (see Figure 1) for our analysis, including interlingual and intralingual comparisons. By comparing the use of metadiscourse markers in Chinese speeches and interpreted language, we aim to unveil the metadiscourse strategies employed by professional interpreters. Simultaneously, the comparison between metadiscourse markers in interpreted language and English speeches serves to identify the distinct metadiscourse features of the interpreted language.

## 5. Data collection and analysis

In this section, we first provide detailed information of the parallel and comparable corpora we have constructed. This is followed by a description of the framework and principle we adopted for the collection of metadiscourse marker data. On top of that, we introduce the tools employed for the statistical process and data analysis.

### 5.1. Corpus construction

The corpus employed in this study is derived from ten international economic conferences held between 2019 and 2021, such as the International Finance Forum (IFF), the Bund Summit, and the Tsinghua PBC School of Finance (PBCSF) Global Finance Forum. The speech topics cover various aspects, such as the new economic development patterns of the global and Chinese economy, international cooperation and global governance, economic recovery in the post-pandemic era, and fintech and digital economy, among others. The speeches at these conferences were delivered in both Chinese and English. Each conference was facilitated by Chinese-English bidirectional simultaneous interpretation, thereby enabling the collection of bilingual data. All the materials used in this study are publicly accessible.

For data selection, we prioritized extemporaneous speeches to ensure the authenticity and representativeness of our corpus. Extemporaneous speeches are speeches that are often well prepared and sometimes even rehearsed in advance, yet delivered with the wording chosen at the moment of presentation, thus allowing for more conversational quality and direct communicative effect than reading from a manuscript, as noted by Lucas and Stob (2019, pp. 234–235). In this study, 96 audio-video clips were collected, including 32 Chinese speeches, 32 corresponding interpreted English versions, and 32 original English speeches. Chinese speeches were delivered by native Chinese speakers, while the corresponding English interpretations were conducted by professional interpreters. The original English speeches, on the other hand, were presented by speakers who utilized English as either their native or working language in the same ten conferences. The data were transcribed, proofread, and aligned, leading to the construction of a Chinese-English Simultaneous Interpreting Parallel Corpus and a Comparable Original English Corpus, totaling about 161,068 tokens. Specifically, the parallel corpus comprises a Source Language Corpus (SLC) in Chinese and a Target Language Corpus (TLC) in English, containing 64 aligned Chinese-English bilingual texts. The comparable corpus refers to the Original English corpus (OEC), which consists of 32 texts of original English speeches. Overall, the corpora consist of speeches derived from real-world simultaneous interpreting scenarios, as opposed to artificially constructed experimental settings, and demonstrate significant homogeneity across various dimensions, including topic selection, temporal span, stylistic genre, linguistic register, and interpreter categories. These features of the corpora ensure the ecological validity of our research.

Table 2 provides a comprehensive overview of the three (sub-)corpora used in this research, including the number of texts, the total number of tokens, the standardized type-token ratio (STTR), and its standard deviation in each sub-corpus. The corpora used in this research show comparability in text types, proportions, and periods (McEnery and Hardie, 2011, p. 20), thereby allowing for the systematic analysis of metadiscourse features in Chinese-English simultaneous interpreting from both intralingual and interlingual perspectives.

**Table 2:** Description of the sub-corpora.

Sub-corpora	Number of texts	Token	STTR	STTR std. dev.
*Parallel corpus*				
Source Language Corpus (SLC)	32	67,859	37.35	61.70
Target Language Corpus (TLC)	32	44,226	36.42	60.25
*Comparative corpus*				
Original English Corpus (OEC)	32	48,983	39.35	57.41

### 5.2. Identification of metadiscourse markers

Cross-linguistic comparative research on metadiscourse presents distinct challenges. Previous researchers have employed strategies such as referencing metadiscourse lists from prior investigations or translating English metadiscourse markers into other languages (Gholami et al., 2014; Siddique et al., 2021, p. 230). In this study, we referred to Hyland’s (2019, pp. 265–272) English metadiscourse inventory and pertinent Chinese metadiscourse studies grounded in Hyland’s framework (Ji, 2011; Li, 2011, 2018). We compiled a comprehensive list of metadiscourse markers suitable for Chinese-English simultaneous interpreting. Subsequently we identified, supplemented, and classified these markers based on the actual context. Just as Hyland (2019, p. xii) emphasized, “whether a linguistic form constitutes a metadiscourse marker depends crucially on the context in which it occurs.”

This highlights the significance of context in distinguishing metadiscourse markers. In ambiguous cases, we relied on the specific context to ascertain the primary function of the marker for proper categorization. Throughout this process, we maintained logical consistency between Chinese and English metadiscourse annotations. We collaboratively undertook the annotation work, arriving at consensus through in-depth discussion and iterative reviews, thereby ensuring the reliability and accuracy of the annotation results.

### 5.3. Data analysis

In the data analysis section, we utilized the Antconc 4.2.0 corpus analysis software to retrieve and statistically analyze the frequency of metadiscourse markers across different corpora. Initially, we computed the frequency characteristics of metadiscourse markers within each corpus. Subsequently, we used a chi-square test and a log-likelihood ratio calculation tool (Liang et al., 2010) to examine the discrepancies in metadiscourse markers among the various corpora. The findings are presented in both raw numbers (raw no.) and standardized frequency (std freq.) formats, with the standardized frequency calculated on a per-thousand-word basis. We set significance levels at 0.05, 0.01, and 0.001 to ascertain the presence of statistically significant variations in metadiscourse markers among different corpora.

## 6. Results and discussion

In general, the Target Language Corpus (TLC) demonstrates the highest frequency of metadiscourse marker usage, amounting to 132.45 tokens per thousand words. This is followed by the Chinese Source Language Corpus (SLC) with a metadiscourse marker usage frequency of 125.95 tokens per thousand words. In comparison, the Original English Corpus (OEC) reveals a relatively lower frequency of metadiscourse markers, with 118.96 tokens per thousand words. Regarding the distribution across the two primary categories of metadiscourse markers, we discern that interactional metadiscourse markers consistently outnumber interactive metadiscourse markers in terms of standardized frequency across all three corpora. Furthermore, our analysis also brings to light significant disparities in the usage frequency of specific metadiscourse markers among the three corpora.

Table 3 illustrates the distribution patterns of various metadiscourse markers across the three corpora based on Hyland’s model. In the following sections we explore the differences of metadiscourse markers used in different corpora from interlingual and intralingual perspectives. Furthermore, we include in our analysis specific examples to shed light on the underlying causes of these discrepancies. The data reveals a high incidence of transitions, self-mentions, and engagement markers across the three corpora, highlighting their crucial role in preserving discourse coherence, molding identity, and fostering audience interaction. In contrast, the usage of endophoric markers appears less frequent. This observation potentially suggests that the content of the corpus tends to convey information directly rather than establishing intricate internal references and associations. This, in turn, hints at a reduced degree of intertextuality.

**Table 3:** Frequencies of metadiscourse in SLC, TLC, and OEC.

	SLC		vs.	TLC		vs.	OEC	
	Raw no.	Std freq.	*p*-value	Raw no.	Std freq.	*p*-value	Raw no.	Std freq.
*Interactive*								
Transitions	2,627	38.71	0.101	1,626	36.77	0.000	1,581	32.28
Frame markers	471	6.94	0.000	433	9.79	0.000	349	7.12
Endophoric markers	112	1.65	0.040	51	1.15	0.048	36	0.73
Evidentials	176	2.59	0.193	134	3.03	0.437	134	2.74
Code glosses	418	6.16	0.001	206	4.66	0.299	205	4.19
Total	3,804	56.05	0.647	2,450	55.40	0.000	2,305	47.06
*Interactional*								
Hedges	604	8.90	0.360	418	9.45	0.726	475	9.70
Boosters	698	10.29	0.000	266	6.01	0.010	363	7.41
Attitude markers	255	3.76	0.551	177	4.00	0.000	343	7.00
Self-mentions	1,106	16.30	0.000	915	20.69	0.001	867	17.70
Engagement markers	2080	30.65	0.000	1,632	36.90	0.000	1,474	30.09
Total	4,743	69.90	0.000	3,408	77.05	0.003	3,522	71.90

### 6.1. Interlingual comparison

By comparing metadiscourse features in the source and target languages of simultaneous interpreting, we can gain deeper insights into the metadiscourse strategies employed by professional interpreters. Statistical data reveals disparities in the frequency and distribution of metadiscourse markers within the parallel corpora. Interpreters made significant adjustments when dealing with frame markers, code glosses, boosters, self-mentions, and engagement markers, while their modifications for transitions, evidentials, hedges, and attitude markers were comparatively conservative. Interpreters used specific strategies in response to different contexts and target audience expectations. These strategies can be summarized into three primary categories: explicitation and simplification, emphasis and weakening, and visibility and invisibility.

In the existing literature, the concepts underpinning these strategies have been discussed across various interpreting modes and language pairs, such as explicitation/simplification (Gumul, 2006; Kajzer-Wietrzny, 2015; Bernardini et al., 2016; Tang and Li, 2016), emphasis/weakening (Pan, 2020; Gu and Wang, 2021), and visibility/invisibility (Angelelli, 2004; Ren, 2010; Ozolins, 2016; Bartłomiejczyk, 2017). As such, these strategies are not entirely novel concepts. However, this research uniquely focuses on how interpreters leverage metadiscourse markers to achieve communicative goals in simultaneous interpreting. While building on existing concepts, this research provides redefinitions and exemplifications of these metadiscourse strategies tailored to the current investigation. The usage of these metadiscourse strategies in interpreting reveals the interpreter’s awareness of the audience and his or her needs for clarification, elaboration, mediation, and interaction.

#### 6.1.1. Explicitation and simplification

Explicitation strategies involve interpreters adding or modifying metadiscourse markers to help the audience understand the logical structure and argument sequence of the source language. Conversely, simplification strategies entail the omission or adaptation of metadiscourse markers to enhance the efficiency of information transmission and the clarity of the target language.

**Table:** 

Example 1	
Source text:	Target text:
**<第一呢 >**, 我们国家的政府非常重视基础建设。 Firstly, our country’s government highly prioritizes infrastructure development.	**<Now>**, **<first of all>**, China pays a great deal of attention to foundational development of the infrastructure.

“Frame markers signify text boundaries or elements of schematic text structure, serving to sequence, label, predict, and shift arguments, thereby enhancing the clarity of discourse for readers or listeners” (Hyland, 2005, p. 51). In this example, the interpreter employs “first of all” to correspond with “第一 (firstly)” in the source text to achieve a sequential function. Additionally, the interpreter introduces “now” to signal a topic shift. Within the given context, this sentence is from a paragraph examining the future trajectory of the digital economy in a speech. Specifically, this sentence initiates the recommendation part of that paragraph. By adding “now,” the interpreter foreshadows an emerging topic or topic branch for the audience, delineating the boundary of the textual structure in the said paragraph and enhancing its logical cohesion. This augmentation potentially aids the audience in comprehending and tracking the paragraph’s viewpoint and arguments.

**Table:** 

Example 2	
Source text:	Target text:
呃, 我也有一些数据啊, 呃, 会呼应这个周院长刚才发布的这个报告。 Uh, I also have some data, uh, will echo the report that Dean Zhou just released.	I will share some figures to echo what Professor Zhou shared**<in the beginning of>** the session.

In this particular instance, the interpreter adds the frame marker “in the beginning of” the session, establishing a connection between the interpreted content and the initial conference session. This action reinforces the intertextuality of the discourse. By adopting the “explicitation” strategy, the interpreter might intend to facilitate the audience in attaining a contextual grasp of the information being conveyed, functioning as a practical guide for tracking and comprehending the entirety of the speech.

**Table:** 

Example 3	
Source text:	Target text:
**<也就是说 >**, **< 比如说 >**, 人民币跨境流动的时候, 人民银行会知道, 其他的央行, 国家的央行是不知道的。 In other words, for example, when the Renminbi is moving across borders, the People’s Bank of China would know, other central banks, countries’ banks would not know.	**<For example>**, the international flow of RMB would be known to PBOC but not to other central banks.

As pointed out by Hyland (2007, p. 284), code glosses carry significant weight in shaping a text’s meaning. They accomplish this by relating a text to its context, while considering the audience’s needs, existing knowledge, intertextual experiences, and relative status. This process involves both exemplification and reformulation. In this example, it is observed that the reformulation code gloss, originally “也就是说 (in other words)” is omitted in the interpreted text, while the exemplification code gloss “比如说 (for example)” is retained. A more in-depth examination of the parallel corpus reveals that this scenario is not an isolated case. Instead, the omission rate of reformulation code glosses tends to be comparatively higher than that of exemplification code glosses in simultaneous interpreting. This disparity could probably be attributed to their distinct functions: reformulation code glosses provide alternative expressions to enhance understanding, making them more prone to omission. Conversely, exemplification code glosses prove more effective in clarifying and explaining complex or abstract concepts, leading to a relatively lower rate of omission.

#### 6.1.2. Emphasis and weakening

Emphasis strategies entail interpreters intensifying or altering metadiscourse markers to bolster proposition certainty and underscore information significance. In contrast, weakening strategies involve reducing or modifying metadiscourse markers. This serves to diminish proposition certainty, promote objectivity, and maintain room for interpretation.

**Table:** 

Example 4	
Source text:	Target text:
本届年会围绕着“后疫情时代:全球治理与国际合作”这一重要的主题开展研讨, 具有很强的现实意义。 This annual meeting focuses on the theme of “Post-pandemic Era: Global Governance and International Cooperation,” which carries significant practical implications.	So this meeting will be centered around the global governance and international cooperation in the post-pandemic era. We **<believe>** it is**<really>** a very timely topic for us to talk about er such a critical period.

In this example, the interpreter adds two boosters, “believe” and “really” to emphasize the importance and timeliness of the conference theme. This strategy is likely used to enhance the expressive quality of the interpreted discourse and convey a stance and attitude that steer the audience toward a deeper understanding and expectation of the theme.

**Table:** 

Example 5	
Source text:	Target text:
**<更重要的>**,**<我觉得><尤其是>**对中国文旅产业,**<更重要的>**是提升创新能力。 More importantly, I believe especially for China’s cultural tourism industry, the more important is to enhance innovation capabilities.	For the Chinese culture and tourism industry, innovation capacity**<should>** be enhanced.

In this example, the attitude marker “更重要的 (more importantly)” and boosters “我觉得 (I believe)” and “尤其是 (especially)” are weakened in the target text, while the engagement marker “should” is added to balance the tonality. The interpreter’s intent here could have been to underscore vital information while achieving an appropriate tonal equilibrium. This is accomplished by tempering and transforming the excessive and repetitive metadiscourse markers found in the source language. In doing so, the interpreter may have conveyed the speaker’s stance and guided the audience’s attitudes, thereby potentially enhancing the effectiveness of information transmission in simultaneous interpreting.

#### 6.1.3. Visibility and invisibility

Visibility strategies refer to interpreters amplifying or modifying metadiscourse markers, thus showcasing their presence and stance, and ultimately elevating interaction and audience engagement. Invisibility strategies, on the other hand, necessitate the reduction or modification of metadiscourse markers, resulting in a diminished prominence of the interpreter and more effective conveyance of the source language’s information and context.

**Table:** 

Example 6	
Source text:	Target text:
呃,<**我们>**这个银行的这个支付功能是商业银行的非常重要的一个功能之一啊。 Uh, the payment function of our bank is a very important function of the commercial banks.	**<You know>**, payment is one of the most important functions of commercial banks.

In this example, the interpreter employs a “visibility” strategy by replacing “我们 (our)” with “you know.” This modification might improve the interaction between the speaker and the audience, involving the audience in the discourse. Engagement markers are classified into five distinct categories: reader mentions, appeals to shared knowledge, directives, questions, and personal asides (Hyland, 2005, p. 182). In this particular case, “我们(our)” from the “reader mentions” category is substituted with “you know,” which falls under the “appeals to shared knowledge” category. Despite this alteration, the interaction between the speaker and the audience remains coherent. The interpreter might have promoted shared knowledge between the speaker and the audience, establishing a foundation for existing information and emphasizing forthcoming content. Additionally, this approach could offer simultaneous interpreters with a temporal buffer, facilitating effective information processing and communication.

**Table:** 

Example 7	
Source text:	Target text:
呃, 正如刚才前面王院长讲的, 我是, 呃,一直以来我是这样一个认知的, 就是<**我们国家>**在互联网数字保险这方面实际上都是走在前面的。 Uh, as just mentioned by Director Wang, I am, uh, always I have been holding such a belief, that is, our country in Internet digital insurance actually takes the lead.	As mentioned by Director Wang, I’ve been holding this thinking that for Internet insurance, <**China>** is leading the world.

In Example 7, the interpreter adopts the “invisibility” strategy by converting “我们国家 (our country)” into “China,” thereby reducing her visibility in the target text. While this approach does somewhat conceal the “self” perspective in the source language, it brings greater clarity to the interpreted text for the international audience, rendering it more transparent and easily comprehensible while maintaining linguistic neutrality. This choice potentially reflects the interpreter’s cultural sensitivity and active commitment to facilitating communication between the speaker and the audience. As corroborated by existing research (Zheng and Ren, 2018; Gu and Tipton, 2020), the interpreter’s role transcends that of a mere observer, functioning as a vital mediator and coordinator.

The discussion above delves into the metadiscourse strategies employed by professional interpreters in simultaneous interpreting, including explicitation/simplification, emphasis/weakening, and visibility/invisibility. It is noteworthy that these strategies do not operate in isolation; rather, they complement one another dynamically, forming synergistic relationships. A multitude of factors might have contributed to interpreters’ strategic choices, including the domain of interpreting, the interpreting scenario, the cultural nuances of the source and target languages, and the expectations of the audience.

### 6.2. Intralingual comparison

In intralingual comparison, we explore the distinctions in metadiscourse features between interpreted text and original English text to shed light on the unique metadiscourse features present in interpreted languages. Statistical findings reveal significant variations (*p* < 0.05) in transitions, frame markers, endophoric markers, hedges, attitude markers, self-mentions, and engagement markers within the intralingual comparison.

Of particular significance are the pronounced discrepancies observed in transitions, frame markers, attitude markers, self-mentions, and engagement markers, which have attained an exceptionally high level of statistical significance (*p* < 0.001). Based on variance analysis and qualitative examination, the study unveils that the interpreted text displays distinct metadiscourse attributes, setting it apart from other forms of discourse. These features predominantly consist of the explicitation of textual logic, objective modal tendency, and audience orientation. A more detailed analysis of each of these features is presented below.

#### 6.2.1. Explicitation of textual logic

In terms of interactive metadiscourse markers, the standardized frequency (55.40) in the TLC stands notably higher than that in the OEC (47.06). More specifically, the employment frequency of transitions, frame markers, and endophoric markers in the TLC significantly exceeds that in the OEC. The variance in the occurrence of frame markers can be ascribed to the interpreter’s deliberate approach to explicate textual structure, thereby enhancing the audience’s grasp and tracking of speech content. Differences in transitions and endophoric markers primarily arise from variances in the content of Chinese and English speeches. Despite these differences, the data suggest that the interpreted language maintains notable features of explicitation of textual logic, which constitutes a critical metadiscourse feature. This finding is in line with Peng (2009), who argues that professional interpreters enjoy distinct advantages in managing global discourse structure. In simultaneous interpreting, a coherent and logical structure in the target text becomes imperative for audience comprehension. Zwischenberger (2010) even posits that “logical cohesion” stands as the paramount criterion for gauging interpreting quality from the audience’s perspective. Consequently, the professional interpreters’ meticulous handling of textual structure in this study reflects their sensitivity to audience needs.

#### 6.2.2. Objective modal tendency

When considering interactional metadiscourse markers, our analysis reveals that the standardized frequency of booster usage in the TLC is 6.01, a figure significantly lower than the observed 7.41 in the OEC. Notably, the frequency of booster usage in the SLC is 10.29, indicating that the reduced frequency of booster utilization does not stem from source language features. Instead, it may result from professional interpreters’ strategic choices to pursue a more objective and neutral mode of expression. This processing strategy highlights the objective modal tendency within the TLC to a certain degree. Both interlingual and intralingual comparisons show no significant differences in the use of hedges, implying that interpreters tend to retain the inherent uncertainty in the source language when handling hedges. This strategy serves to respect and preserve the speakers’ precise rhetorical style while potentially heightening the objectivity of the interpreted language. Moreover, it demonstrates the interpreter’s awareness of linguistic subtleties while maintaining an objective modal tendency.

#### 6.2.3. Audience orientation

In addition to the previously mentioned attributes, it is noteworthy that the standardized frequency of self-mentions (20.69) and engagement markers (36.90) in TLC is significantly higher than that in the OEC and markedly exceeds the figures found in SLC. This finding does not reflect the source language characteristics; rather it illustrates the distinct metadiscourse features intrinsic to the target language. One possible reason for this difference is that simultaneous interpreting necessitates the segmentation and reorganization of the source language information. During this process, interpreters might opt for rendering the subject more explicit to ensure textual coherence, thereby potentially leading to an increased utilization of self-mentions or engagement markers. Another aspect to consider is that interpreters might consciously amplify their employment of self-mentions and engagement markers to enhance interaction and foster audience engagement.

According to Wang (2008), simultaneous interpreters frequently use compression as a linguistic coping strategy in Chinese-to-English simultaneous interpreting. This strategy aims to align with the speakers’ pace of delivery in the specific discourse environments and contexts of simultaneous interpreting, particularly when operating under temporal constraints. Thus, interpreters may find it challenging to succinctly convey every facet of the source language. Furthermore, it is important to recognize that while the overall information in the interpreted language may be more concise than the source language, the degree of simplification applied to self-mentions and engagement markers may be relatively restrained. This phenomenon could contribute to an increase in the frequency of these markers in the interpreted language. Additionally, engagement markers, such as “we,” “you,” and “everyone,” which serve to establish a connection with the audience, as well as directives like “need to,” “must,” “should,” and “take a look,” display a noticeable upswing in interpreted text. The utilization of these markers strengthens guidance, promotes comprehension, and foster audience engagement, thereby contributing to the presentation of audience-oriented metadiscourse features.

### 6.3. Causal explanation

Following a comprehensive analysis of metadiscourse features in Chinese-English simultaneous interpreting from both interlingual and intralingual lenses, we have identified distinct differences in metadiscourse markers across various corpora. These disparities may be attributed to a multitude of factors. In the subsequent sections, we explore the potential causes from the perspectives of contextual factors, source language attributes, and the cognitive psychology of the interpreter.

#### 6.3.1. Contextual factors

Contextual factors encompass elements such as the subject matter and purpose of the speech, the knowledge background and needs of the audience, and the interactive relationship between the speaker and the audience. In economic conferences, speakers typically aim to analyze current economic situations and policies, elucidate economic data and trends, share the latest advancements and discoveries, and offer predictions and recommendations for future economic development. To enhance the persuasiveness of their arguments, speakers may use endophoric and evidentials while also incorporating boosters and attitude markers to capture the audience’s attention and evoke empathy. Given that the majority of the conference attendees are professionals with a solid knowledge background and certain degree of familiarity with the topics under discussion, simultaneous interpreters are likely to refrain from excessive use of code glosses for explanation and clarification. Furthermore, considering that economic conference speeches are frequently accompanied by a wealth of data and charts, interpreters may find it necessary to elevate engagement markers to enhance interaction with the audience and help them better understand and absorb information. Consequently, the differences observed in metadiscourse features in the interpreted language may be heavily influenced by contextual factors.

#### 6.3.2. Source language features

The characteristics of the source language also exert a significant impact on the metadiscourse features in interpreted text, primarily manifesting in the complexity of discourse structure and internal intertextuality. The corpus utilized in this study contains extemporaneous speeches in the economic domain, featuring primary speakers who are experts from government institutions, distinguished professors and researchers in academia, as well as high-ranking executives in the corporate sector. Their speeches exhibit characteristics of spoken language while maintaining certain complexity of written language. Notably they show relatively high proportions of transitions, engagement markers, self-mentions, and boosters.

Furthermore, the discourse reveals considerable intertextuality, as speakers frequently resort to endophoric markers to reference prior content or provide anticipatory cues regarding forthcoming information, assisting the audience in grasping the argument framework. In turn, this process contributes to the establishment of internal logical chains within the discourse, ultimately promoting the audience’s reception and grasp of the conveyed information. Consequently, the complexity and intertextuality inherent in the discourse structure of the source language stand as pivotal factors influencing the metadiscourse features in Chinese-English simultaneous interpreting.

#### 6.3.3. Cognitive psychology of the interpreter

Building on the previous discussion on the interconnected relationship between metadiscourse and metacognition, as well as the statistical distribution of metadiscourse in our research, it is assumed that professional interpreters may use metacognitive regulation to organize, monitor, and adapt their discourse during simultaneous interpreting. Their comprehension, processing, and production of metadiscourse rely on a repertoire of sophisticated cognitive abilities which may impact various facets of metadiscourse marker management. These cognitive faculties include logical reasoning (transitions), discourse structuring (frame markers), intertextual competence (endophoric markers), evaluative judgment (evidentials), predictive interpretation (code glosses), uncertainty moderation (hedges), tone regulation (boosters), emotive recognition (attitude markers), role discernment (self-mentions), and interaction guidance (engagement markers).

In addition, the disparities in these specific capabilities and metacognitive regulation, both within individual interpreters and between different interpreters, could lead to variations in metadiscourse distributions. Consequently, the cognitive psychology of the interpreter constitutes a critical factor in influencing metadiscourse features in simultaneous interpreting. Furthermore, interpreters are expected to employ metacognitive regulation to monitor and coordinate the above-mentioned cognitive capabilities, thereby ensuring the fulfillment of textual and interpersonal functions of metadiscourse in simultaneous interpreting.

## 7. Conclusion

Metadiscourse markers serve as useful tools for interpreters to clarify the source language content and manage the interactive dynamics between the speaker and the audience. These markers play a pivotal role in discourse function and significantly impact the efficacy of communication in interpreting. To comprehensively explore metadiscourse features in Chinese-English simultaneous interpreting, this study employs a mixed-methods approach, leveraging original English speeches as benchmarks. The analysis delves into these features through a composite comparative mode, whereby the source language (Chinese) speeches, the target language versions (interpreted English), and the original English speeches are compared. The findings reveal distinct manifestations of metadiscourse markers in this specific context, furnishing empirical evidence for studies on discourse in interpreting. This investigation represents a step forward in advancing fine-grained analysis in corpus-based interpreting research.

Statistical data reveals significant disparities in the distribution of metadiscourse markers between the source and target languages in simultaneous interpreting, as well as between interpreted English and original English. These disparities may be attributed to a multitude of factors, including contextual factors, source language features, and the cognitive psychology of the interpreter. Professional interpreters harness various metadiscourse strategies (explicitation/simplification, emphasis/weakening, visibility/invisibility) to fulfill both textual and interpersonal roles of the target language, demonstrating a strong metadiscourse awareness. They generally display adeptness in organizing discourse, adapting to contextual nuances, and adjusting language style when processing metadiscourse. By altering the quantity and type of metadiscourse markers, interpreters manage to retain source language information while adapting to the target language features and the audience’s expectations. In comparison with original English speeches, the target language in simultaneous interpreting displays unique metadiscourse features, manifesting explicitation of textual logic, objective modal tendency, and audience orientation. Additionally, this study identifies more entry points for the exploration of cognitive aspects in interpreting studies, for instance, providing insights into how metadiscourse markers may be stored and represented in interpreters’ minds. Moreover, it carries profound implications for future research on interpreting universals, the discourse of interpreting, the cognitive processes of interpreting, interpreter training, and interpreting practice.

It should be noted that this research focused on Chinese-English simultaneous interpreting conducted by professional interpreters and original English speeches within the domain of economic conferences. The extent to which these conclusions can be extrapolated to other language pairs, different interpreting directions, and various settings necessitates further exploration. Moreover, this study employed Hyland’s model as the theoretical framework, demonstrating its adaptability and explanatory power for our research corpus. Future research efforts may choose to continue with or refine this framework to enrich the depth of metadiscourse studies across diverse contexts. To expand the breadth and depth of metadiscourse research in interpreting, researchers may examine and compare various factors in future investigations, such as language pairs, directions and modes of interpreting, subject matter, and interpreter experience and style, among others. Building upon the existing research concerning metadiscourse features in Chinese-English interpreting, future studies could opt to focus on one or several metadiscourse types to delve deeper into the cognitive processing of metadiscourse within the context of consecutive and/or simultaneous interpreting. By establishing a connection between the interpreting process and the interpreting product, we can achieve a more comprehensive understanding of the role and significance of metadiscourse in different modes of interpreting. In the context of future interpreter training, emphasis could be placed on strengthening instruction on metadiscourse markers, fostering the development of cognitive and metacognitive abilities, and guiding students to monitor, evaluate and reflect on their performance during the interpreting process. Such efforts would effectively bolster their metadiscourse competence.

## Data availability statement

The original contributions presented in the study are included in the article/supplementary material, further inquiries can be directed to the corresponding author.

## Author contributions

WR: Conceptualization, Formal analysis, Funding acquisition, Methodology, Project administration, Supervision, Writing – original draft, Writing – review & editing. LW: Conceptualization, Data curation, Investigation, Methodology, Software, Visualization, Writing – original draft.
